# A new look at sodium channel β subunits

**DOI:** 10.1098/rsob.140192

**Published:** 2015-01-07

**Authors:** Sivakumar Namadurai, Nikitha R. Yereddi, Fiona S. Cusdin, Christopher L.-H. Huang, Dimitri Y. Chirgadze, Antony P. Jackson

**Affiliations:** 1 Department of Biochemistry, University of Cambridge, Tennis Court Road, Cambridge CB2 1QW, UK; 2 Physiological Laboratory, University of Cambridge, Cambridge CB2 3EG, UK

**Keywords:** sodium channel β subunits, X-ray crystallography, ion channelopathies

## Abstract

Voltage-gated sodium (Na_v_) channels are intrinsic plasma membrane proteins that initiate the action potential in electrically excitable cells. They are a major focus of research in neurobiology, structural biology, membrane biology and pharmacology. Mutations in Na_v_ channels are implicated in a wide variety of inherited pathologies, including cardiac conduction diseases, myotonic conditions, epilepsy and chronic pain syndromes. Drugs active against Na_v_ channels are used as local anaesthetics, anti-arrhythmics, analgesics and anti-convulsants. The Na_v_ channels are composed of a pore-forming α subunit and associated β subunits. The β subunits are members of the immunoglobulin (Ig) domain family of cell-adhesion molecules. They modulate multiple aspects of Na_v_ channel behaviour and play critical roles in controlling neuronal excitability. The recently published atomic resolution structures of the human β3 and β4 subunit Ig domains open a new chapter in the study of these molecules. In particular, the discovery that β3 subunits form trimers suggests that Na_v_ channel oligomerization may contribute to the functional properties of some β subunits.

## 2. Introduction

**Figure 1.**
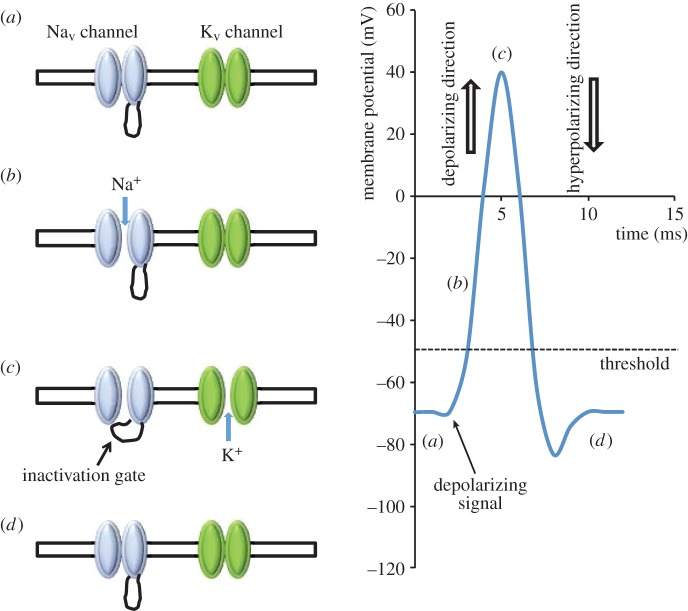
A simplified cartoon showing the main events underlying the action potential. (*a*) Neurons and other electrically excitable cells maintain a plasma membrane resting potential of about −70 mV (the membrane potential is defined relative to the extracellular medium; a potential of −70 mV implies that the cell interior is negative relative to the exterior). The negative resting membrane potential is largely set by the greater membrane permeability of potassium ions compared with that of sodium ions. This occurs in the presence of a high intracellular potassium ion concentration relative to the extracellular media, and a high extracellular to intracellular sodium ion concentration generated by sodium potassium ATPase activity. Under resting conditions, both voltage-gated sodium (Na_v_) and voltage-gated potassium (K_v_) channels are closed. The Na_v_ channels begin to open in response to local membrane depolarization (generated for example by the action of an ionotrophic neurotransmitter on its receptor). (*b*) The net inward flow of sodium ions causes further depolarization that opens more Na_v_ channels, which in turn causes even greater sodium ion entry and further depolarization. This positive feedback ensures the action potential generates a rapid ‘all or nothing’ response, whenever the initial stimulus is above a threshold value (here set at −50 mV). (*c*) In the ‘fast inactivation pathway’, sodium channels enter an inactive state after a few milliseconds, whereby they cannot respond to any further membrane depolarization signals. Fast inactivation is driven by a conformational change in which a hydrophobic sequence (the ‘inactivation gate’) lying within the cytoplasmic loop between domains III and IV moves to block the inner mouth of the pore (see text). As a result, the action potential can only be propagated in the forward direction. The subsequent opening of voltage-gated potassium (K_v_) channels allows potassium ions to move out of the cell, down their electrochemical gradient. (*d*) This moves the membrane potential in the hyperpolarizing direction, restores the membrane to its resting potential and enables the Na_v_ channels to recover from inactivation by returning to their closed conformation.

**Figure 2.**
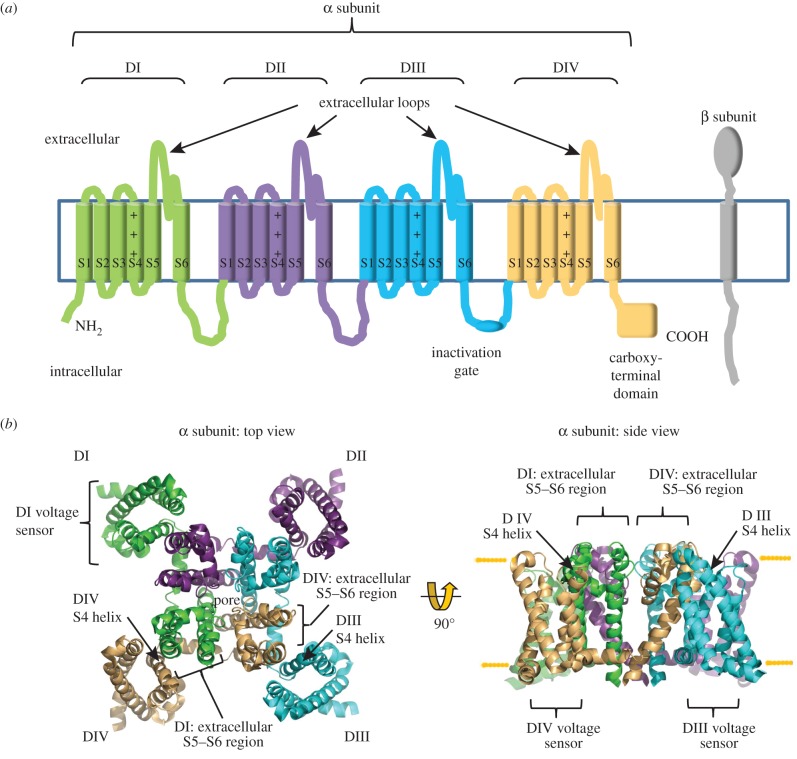
The subunit organization of the voltage-gated Na_v_ channel. (*a*) Cartoon topology of the α and β subunits. Within the α subunit, domains I–IV, the amphipathic helix S4 of the voltage sensors, the extracellular region between helices S5 and S6, the fast inactivation gate and the carboxy-terminus are shown. (*b*) Top view from the extracellular face and side view of the Na_v_ channel from the bacterium *Arcobacter butzeri* (PDB ID code 3RVY). Using this structure as a guide, the inferred position of each vertebrate α subunit domain (I–IV) is shown colour-coded to match figure 2*a*. The location of the voltage sensors with the S4 helix for domains III and IV, and the likely position of the S5–S6 extracellular regions of domains I and IV, are indicated.

Electrically excitable cells such as neurons and myocytes communicate via action potentials, and voltage-gated sodium (Na_v_) channels play an essential role in this process (figure 1). The vertebrate Na_v_ channel α subunit is a single polypeptide chain (molecular mass approx. 260 kDa) that contains the ion-selective component. There are 10 mammalian α subunit genes encoding the proteins Na_v_1.1–Na_v_1.9, and an atypical channel, Na*_x_*. Separate α subunit isoforms are expressed in tissue-specific patterns and exhibit differences in gating behaviour that tailor them for distinct physiological roles [1]. Each vertebrate Na_v_ α subunit contains four homologous but non-identical domains (I–IV), each of which contains six transmembrane helical segments (S1–S6) (figure 2*a*). The domains assemble around the central ion-selective pore [2]. Evidence based on µ conotoxin GIIIA binding suggests that the domains may be organized in a clockwise orientation as viewed from above the extracellular surface [3].

Currently, there are no high-resolution atomic structures of the complete vertebrate Na_v_ channel α subunit. However, the crystal structures of bacterial Na_v_ channels in different conformational states [4–6] (figure 2*b*) and a chimeric voltage-gated potassium (K_v_) channel [7] have been solved. They have provided rich insights into the molecular basis of channel gating. All of the channels with solved structures are homotetramers rather than a single molecule, so they lack the sequence specializations found in the vertebrate Na_v_ channel α subunit, such as the distinct extracellular regions, the intracellular linkers between domains and the unique carboxy-terminus (figure 2*a*). Nevertheless, the basic domain organization of the vertebrate Na_v_ channel is likely to be similar to the subunit organization of the bacterial Na_v_ channels and the K_v_ channel [8]. Using these high-resolution structures as guides, we can say that helices S5 and S6 and the pore loop regions that connect them form the ion-selective pore module. Helices S1–S4 of each domain comprise the voltage sensor and lie on the outer rim of the pore module, at each corner of the α subunit (figure 2*b*). Helix S4 of each voltage sensor is amphipathic, with one face positively charged (figure 2*a,b*). In response to the electric field changes produced by membrane depolarization, helix S4 moves towards the extracellular face of the membrane, thereby initiating conformational changes that open the pore [4,7]. The voltage sensors of domains I–III show the fastest kinetics and allow ion flow to begin [9]. The domain IV voltage sensor responds more slowly. Its movement frees an intracellular linker called the inactivation gate that connects helix S6 of domain III with helix S1 of domain IV (figure 2*a*) [10,11]. As a result, the inactivation gate can now move to occlude the pore and drive the channel into an inactivated state (figure 1). Thus, Na_v_ channel activation and inactivation are structurally, mechanistically and functionally linked [8,12].

Most Na_v_ channels isolated from vertebrate cells contain associated β subunits. There are four β subunit genes (*Scn1b–Scn4b*) encoding proteins β1–β4, respectively [13,14]. Alternative splicing of the *Scn1b* gene adds further diversity to the β1 protein [15]. As with the α subunits, individual β subunits are expressed with distinct tissue specificities [16]. All β subunits are type 1 intrinsic membrane proteins. The extracellular amino-terminal region contains a single V-type immunoglobulin (Ig) domain and a short ‘neck’. This is connected to a single α-helical transmembrane domain and a carboxy-terminal intracellular region (figure 2*a*). However, the primary sequences of the β1 and β3 subunits are more closely related to each other than either is to β2 or β4 [17]. The β1 and β3 subunits are non-covalently bound to the α subunit, but the β2 and β4 subunits are covalently attached to the α subunit via an inter-subunit disulfide bond [18,19].

The β subunits can increase peak current density by increasing the amount of channels in the plasma membrane [13]. They also shift the voltage range over which activation and inactivation occur, and they enhance the rates of inactivation and recovery from inactivation [20,21]. They therefore influence many of the key conformational changes that Na_v_ channels undergo during the action potential cycle [13,16]. Although their gating effects can be subtle, they are clearly important. Mice lacking individual β subunits show a range of isoform-specific pathologies such as epilepsy, ataxia and cardiac conduction diseases. Mutations in the β subunits are associated with a number of human inherited diseases, including epilepsy, neuropathies, cardiac conduction diseases and some types of cancer [13,22].

Site-directed mutagenesis has been used to study the function of β subunits. But these experiments were interpreted using structures inferred from homology modelling [23–25], which is inevitably indirect. However, the structures of the human β3 and β4 subunit Ig domains have recently been solved at atomic resolution by X-ray crystallography [26,27], and should now allow for much clearer functional insights. In this review, we will focus on the new structural determinations, and consider their pathophysiological implications. For a more in-depth discussion of additional aspects of β subunit biology, especially the roles of these molecules in development, signal transduction and their potential as pharmacological targets, we recommend other recent reviews [13,16,22,28].

## 3. The structure of the Na_v_ channel β3 subunit Ig domain and implications for the β1 structure

**Figure 3.**
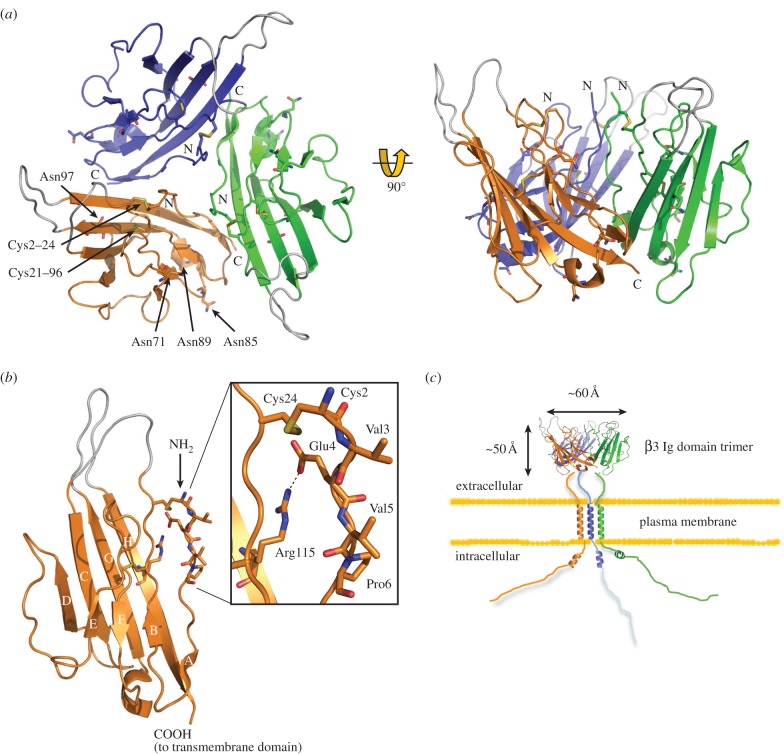
The atomic resolution structure of the β3 subunit Ig domain (PDB ID code 4L1D). (*a*) Diagram showing the arrangement of the β3 trimer. Amino (N) and carboxyl (C) termini are labelled. The Cys21–96 disulfide bond and the Cys2–24 disulfide bonds are labelled on the orange protomer. Potential N-linked glycosylation sites Asn71, Asn85, Asn89 and Asn97 are shown as sticks and labelled for the orange protomer. Loops that are not resolved in the electron density maps due to local disorder are shown in grey at their most probable plausible positions. (*b*) Close-up of the single Na_v_ β3 subunit Ig domain protomer. Residues correspond to loops not visible in the electron density maps due to local disorder are shown in grey. A close-up of the region surrounding the trimer interface is shown in the box. (*c*) Cartoon of the full-length β3 trimer as it may appear on the plasma membrane, with correct dimensions. Note: in these diagrams, the amino acids are numbered from the first residue of the mature protein (i.e. lacking the endoplasmic reticulum targeting signal) [27].

The X-ray structure of the human β3 Ig domain has been solved to a resolution of 2.5 Å. Surprisingly, it forms a trimer in the asymmetric unit (figure 3*a*). This is not a crystal packing artefact: super-resolution imaging detected trimeric full-length β3 subunits as a major species in the plasma membrane of HEK293 cells [27]. Moreover, atomic force microscopy (AFM) imaged full-length β3 subunit monomers, dimers and trimers isolated from transfected cells. The trimer is consistent with previous evidence showing that when expressed in cells, the full-length β3 subunits self-associate in *cis*, and that the Ig domain is necessary for this interaction [25].

The Ig domain is one of the most common protein modules encoded by metazoan genomes, and is found in many immune system proteins and cell-adhesion molecules (CAMs) [29]. It is composed of a two-sheet sandwich of antiparallel β strands which adopt a Greek key topology encasing a disulfide bond-stabilized hydrophobic core. The number and location of the β strands can vary between Ig domains. Loop regions between the β strands often exhibit local disorder when examined in the context of the protein crystal, indicating inherent flexibility [30]. All of these structural features are present in the β3 Ig domain, including a buried disulfide bond Cys21–96 connecting the two faces. However, to promote trimerization, the β3 Ig domain also has a more unusual region of secondary structure. In most Ig domains, the amino-terminal amino acids are held in place by an antiparallel β strand [30]. This feature is absent in β3. Instead, the region is stabilized by a surface disulfide bond (Cys2–24) and a salt bridge between residues Glu4 and Arg115. Consequently, the hydrophobic amino acids Val3, Val5 and Pro6 are forced into a surface-exposed conformation that forms the core of the trimer interface (figure 3*b*). Replacing residue Cys24 with alanine in a C24A mutant prevents the formation of the Cys2–24 disulfide bond and reduces trimer stability, presumably because it interferes with the correct alignment of the trimer interface [27].

The α-helical transmembrane domain of the β3 subunit contains a conserved glutamic acid residue [23]. Peptides encoding transmembrane α-helices with a membrane-embedded glutamic acid readily form dimers and trimers, stabilized by hydrogen bonds between the protonated glutamic acid side chains [31]. We have therefore suggested that the transmembrane glutamic acid residue acts to further stabilize a full-length β3 trimer [27]. A cartoon of the proposed structure for the full-length β3 trimer is shown in figure 3*c*.

**Figure 4.**
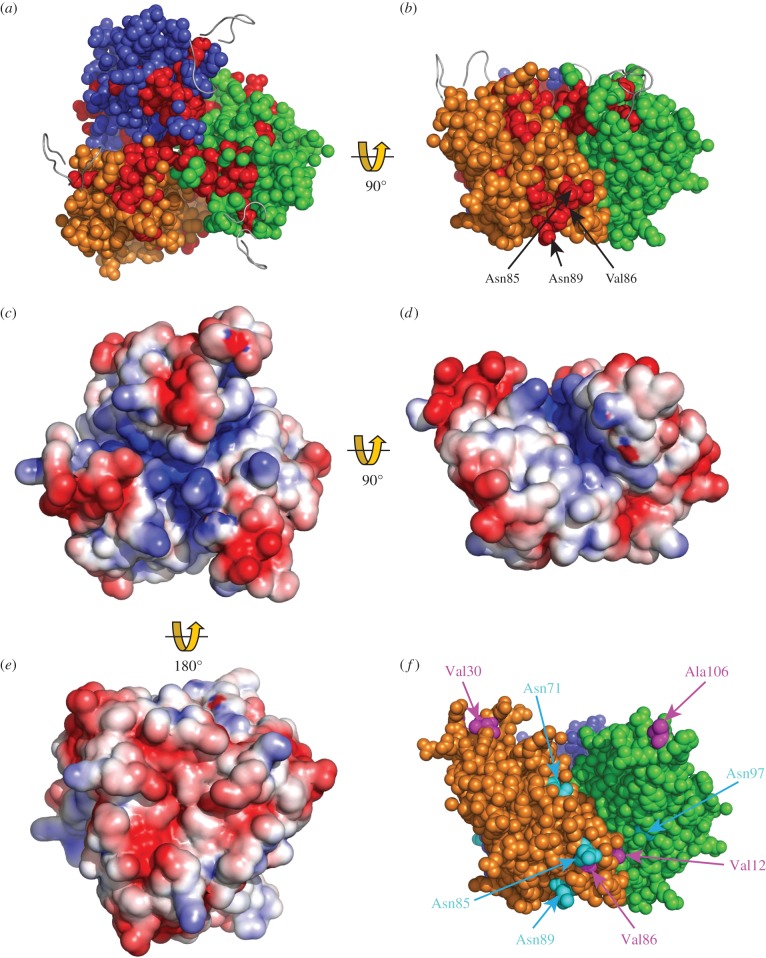
Structural features of the β3 trimer. (*a,b*) Evolutionary trace analysis [25] showing residues in red that are identical between β3 and β1 in all species for which sequences are available, and mapped onto the β3 trimer in (*a*) top and (*b*) side view. The location of putative N-linked glycosylation sites Asn85 and Asn89, and the cardiopathology mutation V86 within a distinct patch of conserved residues (as mentioned in the text), are indicated for the orange protomer. Disordered loops are shown as grey ribbon. (*c–e*) Electrostatic surface potential (ESP) of β3 trimers in (*c*) top, (*d*) side and (*e*) bottom view. ESP was calculated for the trimer surface using the Adaptive Poisson-Boltzmann Solver (APBS) plug-in tool in PyMol [33]. (*f*) The side face of the trimer showing the location of N-linked glycosylation sites (cyan) and the location of separate cardiopathology mutations (purple) mentioned in the text.

Residues between β3 and β1 that are identical in all species can be identified using evolutionary trace analysis [32]. In figure 4*a*,*b*, these amino acids are mapped onto the trimeric β3 Ig domain structure. Most of the trimer interface, including the Cys2 and Cys24 residues, is fully conserved between β3 and β1 [25], as are the surrounding amino acids that help to align and stabilize the trimer interface. This includes an unusual inward-pointing arginine residue (Arg100), whose mutation in β1 is associated with febrile epilepsy [27,34,35]. The β3 Ig domain can interact with β1 when the two are co-expressed in the same cell [25], suggesting that the trimer interface is functionally conserved between β3 and β1. Furthermore, the α-helical transmembrane domain of β1 includes the unusual membrane-buried glutamic acid residue noted above, and it occurs in the same location on the transmembrane domain as with β3 [23,27]. Hence, many of the key features implicated in β3 trimer stability are present in β1. All these factors taken together suggest that the β1 subunit may also assemble into a trimer in a manner broadly similar to β3.

## 4. Insights into Na_v_ channel assembly and α subunit gating from the β3 Ig domain structure

A trimeric β3 structure implies that it may promote the oligomerization of up to three α subunits. Consistent with this, AFM imaging detected heterogeneous complexes containing the cardiac-specific α subunit Na_v_ 1.5 cross-linked by β3 dimers and trimers. The frequency of these α subunit oligomers was reduced to the background level when the Na_v_ 1.5 α subunit was co-expressed with the trimer-disrupting C24A β3 subunit mutant described above [27]. The C24A β3 mutant also attenuated the normal β3-induced gating shift in the voltage sensitivity of inactivation for Na_v_ 1.5 α subunits [20]. Hence the oligomeric status of Na_v_ 1.5 controlled by β3 can affect the electrophysiological properties of the channel.

The β3 subunit is expressed in the heart [36], and it plays an important role in cardiac physiology, because the major pathology associated with the *Scn3b*^−/−^ mouse is spontaneous cardiac arrhythmia [37,38]. The *Scn1b^−/−^*mouse also displays cardiac conduction abnormalities [39]. Evidence that the β1 subunit, like its homologue β3, can cross-link Na_v_ 1.5 α subunits is provided by an interesting study by Mercier *et al*. [40]. Here an Na_v_ 1.5 α subunit mutant associated with the cardiac conduction disease Brugada syndrome (BrS) was retained in the endoplasmic reticulum of cardiomyocytes, leading to reduced peak current. The mutation was also associated with reduced surface expression of the wild-type α subunit, but only if mutant and wild-type α subunits were co-expressed together with β1. Other 1.5 α subunit mutations linked to BrS show similar dominant negative behaviour when co-expressed with the wild-type α subunit in cardiomyocytes [41–44]. Dominant negative phenotypes can often be explained in molecular terms when mutant and wild-type subunits are jointly assembled into a multi-subunit complex, but then every complex that contains any mutant subunit is functionally inhibited. As a result, heterozygous individuals expressing one mutant and one wild-type copy of a protein display a much greater than 50% reduction of the functional protein activity [45]. We suggest that, *in vivo*, individual Na_v_ 1.5 α subunits do not behave as independent molecules, but as oligomeric complexes in which multiple α subunits co-assemble, and which the β3 and β1 subunits may help stabilize. This may help explain why inherited Na_v_ channel cardiopathologies often behave as autosomal dominant phenotypes in pedigree analysis studies [46].

In many—although by no means all—cell-expression systems, the β3 and β1 subunits shift the half maximal voltage *V*_1/2_ for channel activation and inactivation by up to 15 mV in the hyperpolarizing direction (i.e. the voltages corresponding to activation or inactivation of half the channels are displaced to more negative values compared to corresponding values shown by the α subunit alone; the action potential threshold therefore assumes a more negative voltage, leading to an increased likelihood of firing; figure 1). With α subunit partners such as Na_v_ 1.2, 1.3 and 1.5, Ig domains of β3 and β1 are largely responsible for these gating shifts [20,21,23,24,47]. To understand how they might influence gating, we need to consider the structure and location of the β subunit binding site(s) on the α subunits. Unfortunately, the detailed answer to this question is uncertain; even the α : β stoichiometry is not clear. Although the conventional view assumes that individual β subunits bind α subunits at a single site [16], there is no over-riding reason to think that this is correct in all cases. In fact, AFM images show that β3 can bind to Na_v_ 1.5 α subunits at up to four locations around the α subunit [27], probably corresponding to sites on each of the four α subunit domains. On the other hand, the pseudo-symmetry of the vertebrate α subunit domains means that there is no requirement for each binding site to be thermodynamically or structurally equivalent. These experiments were also conducted with a high level of β3 expression, and without the presence of other β subunits, so not all of the potential sites may be occupied by β3 in neurons or myocytes. Nevertheless, they establish the fact that at least under some conditions, a given type of β subunit can bind to the α subunit at multiple locations.

There is currently no molecular information concerning the β3 Ig domain binding site(s) on the α subunit. However, given the structural similarity between the β1 and β3 subunit Ig domains (figure 4*a*,*b*), it is at least plausible that these two closely related β subunits may bind similar or overlapping sites. The intracellular regions of both β1 and β3 do indeed bind to similar sites on the Na_v_ 1.5 α subunit carboxy-terminus [48]. This would indicate that both β1 and β3 can interact with the α subunit close to domain IV (figure 2*a*). Interestingly, the local anaesthetic lignocaine binds to the S6 helix of domain IV [49], and both β3 and β1 attenuate lignocaine binding to Na_v_ 1.3 [50]. A binding site for the β1 Ig domain has been localized to the domain IV extracellular S5–S6 region of the brain-type α subunit Na_v_ 1.2 [51]. In a separate study, two β1-binding sites on the muscle Na_v_ 1.4 α subunit were mapped to the extracellular regions connecting the S5–S6 helices of domain I and domain IV (figure 2*a*) [52]. If the bacterial Na_v_ channel structures accurately reflect the topology of the vertebrate Na_v_ channel, then these two β1 binding sites probably lie about 30–40 Å from each other (figure 2*b*). Since the three-dimensional structure of these α subunit extracellular regions is currently unknown, it is not yet possible to say whether they extend far enough to form a joint binding site. Assuming a top-view clockwise arrangement of the α subunit domains [3], the S5–S6 extracellular region of domain I lies closest to the voltage sensor of domain IV, while the S5–S6 extracellular region of domain IV abuts the voltage sensor of domain III. If β1 is like β3 and forms a trimer, then viewed from above, its shape will be an approximate equilateral triangle of side length about 60 Å (figure 3*c*). Based on the dimensions of the bacterial channel, adjacent voltage sensors will be about 60–70 Å apart. Hence, the Ig domains of β1 (and β3) at this site could lie close to the α subunit voltage sensors of both domains IV and III. How then could they influence voltage gating sensitivity?

A hyperpolarizing shift could occur if a negatively charged protein is brought sufficiently close to a voltage sensor so that it generates screening surface charge [53–56]. The calculated isoelectric points of the β1 and the β3 Ig domains are 4.95 and 5.28, respectively, so they will both carry a net negative charge at pH 7.4. Interestingly, the electrostatic surface potential of the β3 trimer suggests a dipole-like quality. Positive charges are concentrated on the top surface and the clefts between protomers, although the loop regions at each trimer vertex are negatively charged (figure 4*c*,*d*). By contrast, the trimer underside presents negative charges concentrated within a centrally located, shallow concave face (figure 4*e*). The full significance of this curious feature will remain unclear until the atomic resolution structure(s) of the β3-binding sites on the α subunit are determined. But it is consistent with a role in presenting negative charge to the α subunit voltage sensors.

An important consideration is that, *in vivo*, the β subunit Ig domains are heterogeneously glycosylated [25,57]. Asparagine (N)-linked glycosylation sites are defined by a conserved Asn-X-(Ser/Thr) motif, in which X can be any amino acid except proline [58] and mature N-linked sugar residues often contain sialic acid moieties on their terminal branches [59]. When tested in CHO cells with Na_v_ 1.2, 1.5 and 1.7, the hyperpolarizing gating shifts induced by β1 were abolished, both by mutagenic removal of the N-linked sites and by using a cell-line lacking sialyl transferase [60]. This indicates an important role for sialylation of the β1 Ig domain N-linked glycosylation sites in modulating the gating voltage shifts. The same post-translational modifications will undoubtedly occur on the β3 Ig domain. There are four potential N-linked glycosylation sites per β3 Ig domain protomer, and all of them point outwards from the surface-exposed faces of the trimer [27] (figure 4*f*). The potential N-linked glycosylation sites Asn85 and Asn89 are particularly interesting. They are fully conserved between β3 and β1 in all known species (figure 4*b*), as are the immediately surrounding residues Val86, Thre87 and Gly92 [25,27]. Furthermore, these two N-linked glycosylation sites lie close to two amino acids that are separately mutated in different inherited cardiopathologies: V86I [61] and V12M [62] (figure 4*f*). Residue V12 normally forms a hydrophobic contact with Leu116 of an adjacent protomer that helps maintain the correct trimer organization [27]. The V12M mutation probably destabilizes this interaction. Two further mutations in the β3 Ig domain that are associated with inherited cardiopathologies have been described. These are A106V [63] and V30G [64], and occur on adjacent Ig domain loops (figure 4*f*). In the crystal structure, the conformation of these two regions is unresolved due to local disorder (figure 3*a*,*b*) [27]. In other Ig domains, the equivalent loop regions can act as binding sites for interacting proteins. For example, these regions of antibody Fab fragment Ig domains constitute the antigen-binding site [30]. In β3, their flexible nature, outwardly pointing locations and associations with cardiopathologies make them plausible candidates for an α subunit binding site—perhaps interacting with the S5–S6 extracellular regions of domains I and IV (and see also §5).

We suggest that the hyperpolarizing shifts in voltage gating shown by the β3 and β1 subunits are caused by an electrostatic mechanism in which the Ig domain is held in place via binding to extracellular regions of the α subunit. This would position the Ig domains so as to present negative charges from sialic acid residues (and perhaps the proteins themselves), close enough to one or more voltage sensors to affect gating behaviour, as previously proposed [55,56,60]. It should be noted, however, that in some other expression systems the same β subunits can produce *depolarizing* shifts in the *V*_1/2_ values for activation and inactivation [65–67]. Furthermore, the magnitude of the shifts can vary between α subunit partners. For example, it has been reported that β3 subunits have little or no effects on the gating voltage sensitivity of Na_v_ 1.8 and Na_v_ 1.6 [22,68]. It is hard to see how such differences can arise, other than by variations in post-translational modification and/or cell-specific and isoform-specific differences in the precise molecular organization of the subunits within the channel complex. An additional factor may be the wider molecular environment of the channel. The Na_v_ channels do not exist in isolation on the plasma membrane, but as part of local protein clusters that include other ion channels and CAMs [69]. The influence of this clustering on β subunit-induced gating shifts remains to be characterized.

The β3 and β1 subunits can also enhance the rate of inactivation and recovery from inactivation, and increase the fraction of channels that gate in a fast-acting mode [23,70,71]. Within the α subunit, the carboxy-terminal domain binds to the inactivation gate, and this interaction stabilizes fast inactivation [72]. The α subunit carboxy-terminal domain also binds to the intracellular region of β3 and β1. An epilepsy-associated mutation in the carboxy-terminal domain of Na_v_ 1.1 disrupts this interaction and attenuates the normal β1-induced enhancement of inactivation [48]. This suggests that these β subunits may facilitate fast inactivation by binding to, and optimally aligning the complex between, the α subunit carboxy-terminal domain and the inactivation gate. NMR studies on the intracellular region β3 intracellular region indicate that it is largely disordered, but with a short juxtamembrane sequence that can adopt a negatively charged amphipathic α-helix [65] (figure 3*c*). The intracellular regions are long enough to form a complex with the α subunit carboxy-terminus and the inactivation gate. But the formation of α subunit oligomers via β3- and β1-induced cross-linking could greatly facilitate and stabilize these interactions. The Na_v_ channel oligomer would then behave as an integrated allosteric protein [73]. Consistent with this proposal, it has been shown that mutational disruption of the β1 region equivalent to the β3 trimer interface reduced the fraction of Na_v_ 1.2 that acted in the fast-gating mode [74].

## 5. The β4 Ig domain structure and functional implications

**Figure 5.**
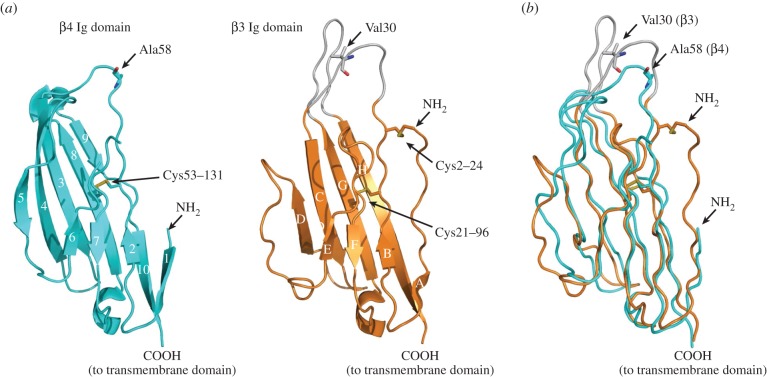
The atomic resolution structure of the β4 subunit Ig domain. (*a*) The β4 Ig domain (PDB ID code 4MZ2) and comparison with similar orientation with the β3 Ig domain. (*b*) Supposition of the β4 Ig domain (cyan) and a single β3 Ig domain protomer (orange), showing the location of the Ala58 residue (corresponding to the free Cys58 residue of β4) and the Val30 residue of β3 mentioned in the text.

The structure of the human β4 Ig domain has been solved at 1.7 Å resolution [26]. Overall, the Ig domains of β4 and a β3 protomer are remarkably similar, despite their low level of sequence similarity (figure 5*a*,*b*). However, there are also some striking differences. The most obvious is that the β4 Ig domain is a monomer in the crystal asymmetric unit. There is currently no evidence that β4 forms *cis* homodimers or homotrimers *in vivo*. Unusually for an Ig domain, the first seven amino-terminal residues (which in β3 comprise the trimer interface) are disordered and unresolved in the β4 Ig domain crystal structure. There are also significant sequence differences in this region between β4 and β3. In particular, the C2–24 disulfide bond is not present in β4, because the equivalent cysteine residue at position 2 is not present. The Cys24-equivalent residue is present in β4 as Cys58, but to avoid potential complications caused by the presence of a free cysteine, this residue was mutated to alanine in the protein used for crystallization. The Cys58 residue forms a disulfide bond with the α subunit *in vivo* [18], and so must be located within an α subunit binding site of the β4 subunit. Residue 58 is surrounded by side chains of bulky hydrophobic amino acids—particularly Phe59, which is exposed and outward-pointing in the crystal [26], but which is probably buried into a suitable hydrophobic pocket on the α subunit when correctly assembled *in vivo*. Interestingly, residue 58 is located on one of the β4 Ig domain surface loops, although the region appears notably less flexible than the corresponding loop in the β3 protomer (figure 5*a*) [26]. When the β4 and β3 structures are overlaid (figure 5*b*), the region of β4 surrounding residue 58 directly corresponds to the loop region of β3 that contains the V30G cardiopathology mutation noted above (figure 4*f*). This provides some additional evidence that the loop regions of the β subunit Ig domains may be α subunit binding sites (see §4).

In common with most Ig domains, the β4 structure contains a buried disulfide bond (C53–131) (figure 5*a*) [30]. The conventional view has been that the disulfide bond stabilizes Ig domain folding by covalently connecting the two β sheet faces. Surprisingly, however, a C131W mutation still folded and was efficiently exported to the plasma membrane through the secretory pathway. Rather than completely disrupting folding, X-ray crystallographic analysis revealed that the C131W mutation had a more subtle and interesting effect. Breaking the internal disulfide bond led to a local remodelling of the β sheets surrounding the Cys58-containing loop region [26]. This work has important implications for other β subunits. In particular, an equivalent mutation (C121W) in β1 causes generalized epilepsy with febrile seizures plus (GEFS+). Here, the mutant β1 subunit can no longer bind to and modulate the brain-specific Na_v_ 1.2 α subunit [75]. It is therefore probable that in β1, the C121W mutation selectively perturbs an α subunit interaction site on the β1 Ig domain loop region.

The β4 binding site(s) on the α subunit are unclear. However, one clue comes from the ability of β4 to inhibit toxin-binding. For example, co-expression of β4 with the Na_v_ 1.2 α subunit reduced the affinity of the tarantula toxin ProTx-II for the channel, and β4 also reduced sodium influx in Na_v_ 1.2 when tested with the scorpion toxin TsVII [26]. The ProTx-II toxin binds to the α subunit voltage sensors of domains I, II and IV, and TsVII binds predominantly to the α subunit voltage sensor of domain II [10]. On this basis, it has been suggested that β4 may influence the domain II voltage sensor [26]. This presumed β4 binding site on the α subunit is different from the one known β1 binding site noted above, and Na_v_ 1.1 channels can be isolated containing both non-covalently associated β1 and covalently bound β4 subunits [76]. On the other hand, under conditions of high expression, β4 can partially (but not completely) displace β1 from Na_v_ 1.1 [76]. Hence, a more subtle possibility is that the β4 and β1 subunits may bind at more than one site on this α subunit, some of which are overlapping. This is reminiscent of the multiple β3 binding sites on Na_v_ 1.5 detected by AFM and noted earlier. If so, then there are some interesting implications. In neurons, expression of the α and β subunit isoforms can change significantly, both during development [77] and in some pathologies such as diabetic neuropathy [78] and neuropathic pain [79]. If β3 and β1 can induce α subunit cross-linking, while monomeric β4 cannot, then varying expression of different structural classes of β subunits may differentially modulate the oligomeric state of the channels, and this will contribute to both structural and functional heterogeneity within the Na_v_ channels on the plasma membrane.

A unique aspect of β4 behaviour is its ability to promote ‘resurgent current’. This phenomenon occurs when channels are opened by membrane depolarization but are rapidly blocked by an endogenous protein that inhibits current flow, yet also prevents binding of the fast inactivation gate to the pore. Following membrane repolarization, the blocker is removed, leading to a brief resurgent current flow before the channels inactivate via the conventional pathway [80]. The blocking factor has been localized to sequences within the β4 intracellular region [81]. As with β3, the secondary structure of the β4 intracellular region is predominantly disordered, but with a short segment adjacent to the membrane that displays amphipathic α-helical potential—although in the case of β4, the charges along one face of the helix are positive. It is these charges that are critical for the resurgent current effect [82]. Disordered regions of proteins can sample many different conformations simultaneously, and can provide high-specificity binding even with low intrinsic affinity [83]. They may be advantageous in situations encountered by the intracellular regions of both β3 and β4, with their requirement for different binding interactions occurring at specific parts in the channel gating cycle.

The β4 subunit is closest in sequence identity to β2. The β2 subunit also contains a free cysteine at the equivalent position to Cys58, and likewise forms a disulfide bond with the α subunit *in vivo* [19]. However, it is not known if β2 and β4 share overlapping binding sites. For Na_v_ 1.5, the β2 subunit induces hyperpolarizing gating shifts that are sialic-acid-dependent [84]. On the other hand, the β2 subunit induces sialic-acid-independent depolarizing gating shifts for Na_v_ 1.2 [84]. There are currently no structural explanations for these strikingly disparate effects. Again, they emphasize the complex isoform-specific nature of the binding interactions between Na_v_ α and β subunits. Mice lacking β2 expression have a relatively mild phenotype, although they are more prone to seizures. Interestingly, these mice display a significantly reduced Na_v_ channel density in some classes of neurons, especially in the hippocampus [85]. It is therefore possible that β2 has a particularly important role in stabilizing Na_v_ channels in the plasma membrane [86].

## 6. The Na_v_ β subunits as cell-adhesion molecules

The Na_v_ β subunits are related to members of the CAMs superfamily [17], and there is growing evidence that β subunits display CAM-like behaviour both in *cis* and in *trans*. The β1 subunit Ig domain can interact in *cis* with other CAMs such as neurofascin-155 and neurofascin-186, contactins and cadherins, and β3 can bind neurofascin-186 [87,88]. The β1 and β2 subunits interact with extracellular matrix proteins such as tenascin-R that are secreted by oligodendrocytes during myelination [89]. These multiple interactions all help localize Na_v_ channels to discrete clusters at the nodes of Ranvier in myelinated neurons, and probably increase the total amount of Na_v_ channels in the membrane.

**Figure 6.**
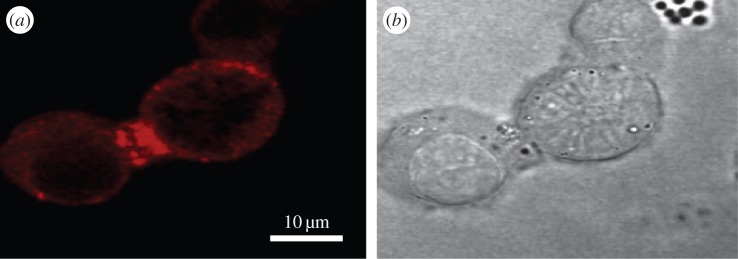
The rat neuronal-like cell-line PC12 expresses the β3 subunit endogenously [23]. (*a*) β3 accumulation at the contact site between two PC12 cells, as detected by immunofluorescence microscopy, and (*b*) DIC image of the same cells. Cells were stained with an affinity-purified rabbit polyclonal antibody raised against a β3-specific peptide corresponding to the intracellular region of the molecule. The antibody has been described previously. It detects β3 in both Western blotting and immunohistochemistry experiments, and its binding is inhibited by prior incubation with the immunizing peptide [92]. The PC12 cells were fixed, permeablized and stained as described previously [93].

The Na_v_ β subunits also act as *trans*-binding CAMs independently of α subunits. This has been conveniently studied by expressing them in the *Drosophila* S2 cell-line and monitoring cell adhesion visually. Here, the β1 and β2 subunits promoted cell adhesion [90], while the β3 subunit did not [91]. On the other hand, β3-mediated cell adhesion was detected when assayed in more physiologically mammalian cell-lines, both by immunoprecipitation [25] and by immunofluorescence (figure 6). The *trans* cell adhesion of β3-subunits required an intact Cys2–24 disulfide bond [25]. Hence, the *cis*-formed β3 trimer is probably needed to form the *trans*-binding cell–cell contacts. This is similar to the behaviour shown by the CAM myelin P0 which stabilizes the myelin sheath around peripheral neurons [94]. The myelin P0 molecules assemble as tetramers in *cis*, and then the tetramers associate in *trans* to form tight intercellular contacts [94]. It may be significant that myelin P0 is a distant paralogue of the Na_v_ β subunits, and its Ig domain has previously been used to model both the β1 and β3 Ig domains [23,25,74,95].

The demonstration that at least some β subunits have both *cis* and *trans* CAM activity complicates the interpretation of knockout mouse phenotypes. For example, rapid conduction in the heart requires the cardiomyocytes to be electrically coupled to each other via their apposed intercalated discs [46,96]. The β subunits are located on the intercalated discs of ventricular cardiomyocytes [38,97,98], and their absence will probably have multiple effects: the Na_v_ channels will display altered gating behaviour and their location at the intercalated discs may be destabilized, but cell adhesion between cardiomyocytes could also be compromised, potentially leading to loss of signal cohesion during the intercellular propagation of the cardiac action potential. We should be open to the possibility that some of the pathologies shown by β subunit knockout mice (and by the human pathologies associated with β subunit mutations) reflect abnormalities in cell adhesion rather than direct electrophysiological effects on the Na_v_ channels themselves.

## 7. Summary

An earlier review described Na_v_ channel β subunits as ‘anything but auxiliary’ [99]. We agree with this sentiment, and we emphasize the integration of the β subunits with other Na_v_ channel components. As a case in point, we note the current interest in the pharmacological potential of animal toxins that target Na_v_ channels [100]. In some examples, the binding affinity of these toxins for Na_v_ channels is actually *increased* by the presence of specific β subunits [101,102]. This raises interesting questions about the nature of the toxin-binding sites. The important point is that these toxins evolved to act against Na_v_ channels in their normal physiological context, which includes the β subunits. We suggest that screening assays for such toxins and drugs should include, wherever possible, the relevant β subunit(s) for the Na_v_ channels in question.

These are interesting times for Na_v_ channel β subunit research. The new results from X-ray crystallography and molecular imaging provide the first detailed look at the molecules, and will encourage both the generation of detailed functional hypotheses and their experimental testing.
